# Middle ear squamous papilloma: A report of four cases analyzed by HPV and EBV *in situ* hybridization

**DOI:** 10.3892/ol.2013.1675

**Published:** 2013-11-11

**Authors:** HAN ZHOU, ZHIBIN CHEN, WEIMING ZHANG, GUANGQIAN XING

**Affiliations:** 1 Department of Otolaryngology, The First Affiliated Hospital, Nanjing Medical University, Nanjing, Jiangsu 210029, P.R. China; 2 Department of Pathology, The First Affiliated Hospital, Nanjing Medical University, Nanjing, Jiangsu 210029, P.R. China

**Keywords:** human papilloma virus, Epstein-Barr virus, middle ear, squamous papilloma, *in situ* hybridization

## Abstract

Squamous papilloma involving the middle ear as a primary lesion is an extremely rare occurrence. The aims of the present study were to investigate the presence of human papilloma virus (HPV) and Epstein-Barr virus (EBV) infections in primary middle ear squamous papilloma and to describe the clinical and pathological features of the disease along with therapeutic strategies. A retrospective review was conducted of four patients with clinical and pathological diagnoses of middle ear squamous papilloma. *In situ* hybridization (ISH) for a wide range of HPV DNA subtypes and EBV-encoded RNA was performed in the tissue samples obtained from these patients. Only two cases of primary squamous papilloma in the middle ear have been previously reported in the English literature. These papillomas developed in males of ~60-years of age and otorrhea was the most frequent complaint. Premalignant changes were observed in two of the present cases and ISH of HPV and EBV was negative in all four cases. The results of the present study indicated that chronic inflammatory stimulation, not HPV and EBV infection, is involved in the occurrence of middle ear squamous papilloma and its malignant transformation. Radical surgery and long-term postoperative follow-up are recommended due to its malignant and recurrent potential. Further genetic investigations with additional new cases are required to clarify the pathogenesis of squamous papilloma involving the middle ear.

## Introduction

Squamous papillomas are benign epithelial tumors that occur on the skin of the face and body and most often appear in the mouth or genital regions. Squamous papilloma involving the middle ear as a primary lesion is an extremely rare occurrence (1). Few cases have been previously reported in the English literature (2), hence, its etiology and clinicopathological features remain unclear. Specific hypotheses indicate that squamous papilloma lesions correlate with viral infection, chronic inflammation, allergies or environmental pollutants. Previous studies have shown that infection with human papilloma virus (HPV) is involved in the occurrence of papillomas in the head and neck region (3–6) and it is known that Epstein-Barr virus (EBV) is carried by ~90% of the adult population worldwide as a lifelong asymptomatic infection (7).

Although no causal correlation has been established between viral infections, including HPV and EBV, and the development of middle ear squamous papilloma, it remains a possibility that requires important consideration. The current study reported four cases of primary middle ear squamous papilloma and the results of HPV and EBV *in situ* hybridization (ISH). The pathogenesis and diagnostic, therapeutic and prognostic aspects of this tumor are also discussed in hope that the results of the present study are useful for clarifying diagnostic and therapeutic strategies for this type of papilloma and the involvement of HPV and EBV infections.

## Materials and methods

### Patients

Between 2009 and 2012, four patients were treated at the First Affiliated Hospital of Nanjing Medical University (Nanjing, China) with an initial pathological diagnosis of squamous papilloma of the middle ear. The records of these four patients were retrospectively reviewed, including the clinical history, treatment, follow-up, radiological data and pathology reports. Paraffin-embedded tissue blocks from the middle ear of these patients were recovered, sectioned and stained with hematoxylin and eosin. To avoid interobserver variations, two pathologists reviewed all pathological slides and were in agreement with the final pathological reports. All lesions in this study were associated with the middle ear and there was no evidence of prior papillomas in the external auditory meatus or nasopharynx. The current study was approved by the Institutional Review Board of the First Affiliated Hospital of Nanjing Medical University. Written informed consent was obtained from the patients.

### ISH for HPV DNA

For detecting the presence of HPV, ISH was conducted with a wide-spectrum digoxigenin-labeled probe (Triplex International Biosciences Co. Ltd., Fuzhou, China) for common HPV types according to the manufacturer’s instructions. The wide-spectrum probe targets the genomic DNA of HPV types 5, 6, 8, 11, 16, 18, 26, 27, 30, 31, 33, 35, 39, 40, 41, 42, 43, 45, 47, 48, 51, 52, 53, 54, 55, 57, 58 and 59. Sections from the tissue blocks were deparaffinized and rehydrated in graded alcohols and distilled water. Target sample pretreatment was performed in a high-power microwave oven. The hybridization reaction was detected by incubation with an anti-digoxigenin antibody tagged with horseradish peroxidase (POD), and diaminobenzidine (DAB) was applied as the chromogen. Slides were counterstained with hematoxylin and appropriate positive and negative controls were included in each assay. Positive staining was defined as the presence of dark brown granules in the nuclei of epithelial cells at the site of hybridization.

### ISH for EBV-encoded RNA (EBER)

EBER was detected by ISH with the EBER Detection kit (Triplex International Biosciences Co. Ltd.) according to the manufacturer’s instructions. Next, the sections were deparaffinized, rehydrated and predigested with proteinase K and a hybridization solution containing the digoxigenin-labeled EBER nucleic acid probe was applied. Detection of the hybridized probe was performed by application of anti-digoxigenin-POD and the coloring reaction was performed with DAB. Sections were counter-stained with hematoxylin and brown nuclear staining was regarded as a positive hybridization signal.

## Results

### Clinical data

**Figure 1:**
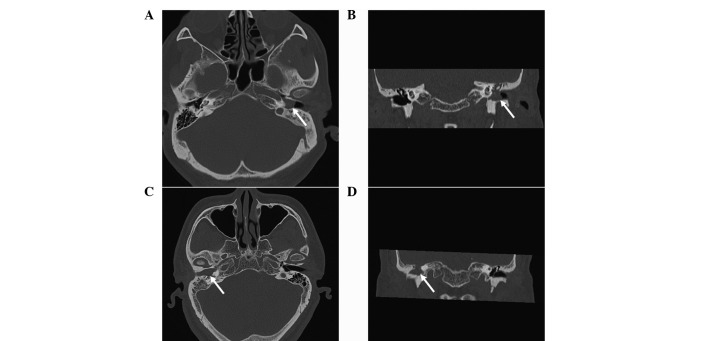
(A and B) High-resolution axial and coronal CT scans from case 2 showed that soft tissue was present in the middle ear region on the left side and involved the ossicular chain without structural or morphological modifications, as indicated by the white arrows. (C and D) Axial and coronal CT scans from case 4 showed that soft tissue density was evident in the right side of the middle ear and in the sclerotic mastoid, with extension into the external auditory meatus, as indicated by the white arrows. CT, computed tomography.

The clinical data of the four patients are summarized in Table I. All patients were male with ages ranging between 29 and 70 years, with variable courses of disease ranging between 2 months and 50 years. Otorrhea was the most frequent complaint, occurring in three of the four patients. Other symptoms, including hearing loss (n=3), otalgia (n=3), tinnitus (n=2) and aural fullness (n=2), were also observed. All subjects received computed tomography (CT) scans of the temporal bone prior to surgery and these scans showed soft tissue density between all regions of the middle ear and the mastoid with or without extension into the external auditory meatus (Fig. 1). Mild bone erosion of the promontory was observed on the CT scan of case 1.

Clinical management of these patients was based primarily on the preoperative imaging observations and intraoperative frozen-section examinations. Papillomas associated with premalignant changes were found in the frozen sections of cases 1 and 2 and in these cases, extended resections of the lesion (subtotal or partial temporal bone resection) had been performed. Radical tympanomastoidectomy was used in the other two cases to guarantee radical resection of the neoplasms. All patients recovered well postoperatively and were discharged as scheduled. To date, the patients have been followed for between 6 and 36 months without evidence of recurrent disease, clinically and radiologically.

### Pathological observations

**Figure 2:**
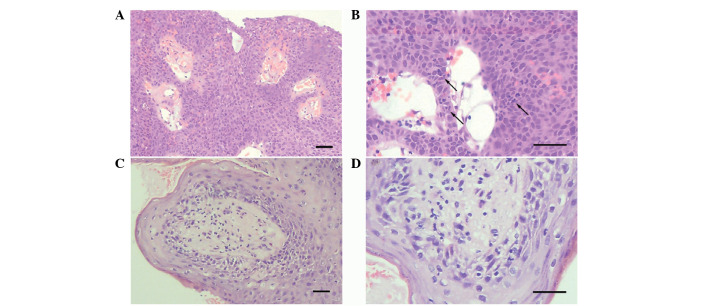
Representative histopathology of squamous papillary lesions (hematoxylin and eosin staining; scale bar, 50 μm). (A) A section from case 2 showed exophytic papillary projections with fibrovascular cores covered by keratinizing squamous epithelium (magnification, ×100). (B) Higher magnification of case 2 showed focal high-grade cellular dysplasia in the basal layers of squamous epithelium, as indicated by the black arrows (magnification, ×200). (C and D) A section from case 4 showed squamous papilloma with normal maturation epithelium. (C) Magnification, ×100; (D) magnification, ×200.

Histological examination of these cases confirmed the diagnosis of squamous papilloma. In addition, premalignant changes, including carcinoma *in situ* and high grade squamous intraepithelial neoplasia, were observed in the sections of cases 1 and 2. Representative histopathological images of the middle ear squamous papillomas and premalignant changes are shown in Fig. 2. Squamous papilloma exhibits as fibrovascular axes covered with pluristratified keratinized epithelium and a clear margin with no cytological features of malignancy in exophytic papillary projections. Premalignant change is shown by high-grade dysplasia of the epithelial cells without penetration through the basement membrane in papillary fronds.

### HPV and EBV detection

**Figure 3:**
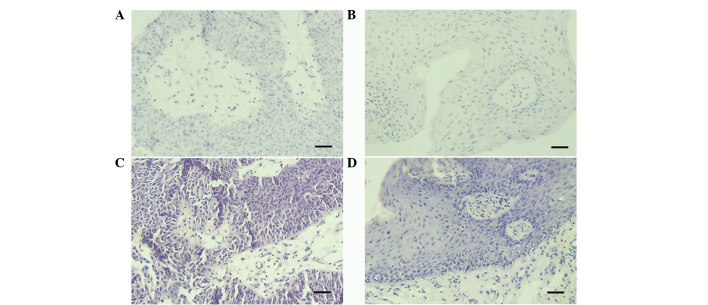
ISH for HPV showed no detectable HPV-types in the sections from cases (A) 2 and (B) 4. ISH for EBV-EBER was negative in the sections from cases (C) 2 and (D) 4 (Scale bar, 50 μm). ISH, *in situ* hybridization; HPV, human papilloma virus; EBV, Epstein-Barr virus; EBER, EBV-encoded RNA.

HPV and EBV ISH showed no detectable HPV or EBV genomes in any of the four cases with middle ear squamous papilloma (Fig. 3).

## Discussion

Squamous papilloma may, in theory, occur anywhere on the epidermis and mucosa, but in the head and neck region, it is most commonly found in the mouth and throat. Primary middle ear squamous papilloma is an exceedingly rare temporal bone neoplasm and an Ovid Medline search using the search terms ‘squamous papilloma’ and ‘middle ear/temporal bone’ returned only two cases in the English literature (2,8). The data in the previous literature consisted only of case reports and the clinical features and pathogenesis of the tumor are vague due to the low frequency of the disease. The present study presented four patients with primary middle ear squamous papilloma. Premalignant changes were observed in two of these cases and the results of ISH showed that HPV and EBV are not present in these lesions. Based on these results, we hypothesize that HPV and EBV infection may not play a role in the pathogenesis of middle ear squamous papilloma.

The etiology and pathogenesis of papillomas of the middle ear remain unknown. CT scans of the current patients did not detect lesions in the sinonasal cavity, nasopharynx or pharyngeal opening of the auditory tube. In addition, none of the patients had a past history of papillomas of the external ear canal or relevant surgical procedures. Thus, this infers that the tumors in the present cases did not arise in the adjacent area and become incidentally involved with the middle ear via the eustachian tube or the external acoustic meatus. Therefore, this indicates that the tumors were of multicentric primary origin. Furthermore, inverted papilloma, an additional type of papilloma, rarely occurs in the middle ear (9). Ectopic migration of ectodermal tissue to the middle ear, viruses, chronic inflammation, allergies and carcinogenic exposure have all been considered as possible etiological factors for this neoplasm (9,10). Thus, it is reasonable to conclude that these factors may also be potential causes for the onset of squamous papilloma in the middle ear. Among these factors, viral and HPV infections, in particular, have long been considered to stimulate the development of squamous papilloma in the squamous epithelial cells of the skin.

There is growing evidence to support the association of HPV with the pathogenesis of squamous papillomas, including genital warts and recurrent childhood respiratory papillomatosis (11,12). HPV encompasses a group of double-stranded DNA viruses of the papovavirus subgroup A and a number of HPV subtypes are associated with lesions of the head and neck. High-risk HPV has been identified in middle ear carcinomas (13), but the correlation between HPV infection and papillomas in the temporal bone remains unknown. We hypothesize that HPV infection is involved in the occurrence of middle ear squamous papilloma in a manner similar to such tumors in the genital regions. Detection of viral presence by ISH has demonstrated a valuable method for determining diagnoses and has led to new insights into viral and neoplastic diseases (14,15). In the current study, a wide-spectrum probe for HPV DNA was applied to paraffin-embedded papilloma tissues, but all four cases of squamous papilloma were found to be HPV-negative.

The HPV infection rate in middle ear squamous papilloma has not been previously reported and only a few studies have investigated the presence of HPV infection in middle ear inverted papilloma. Previous attempts to identify HPV in the tissue sections of seven cases of primary temporal inverted papilloma have been described in previous studies (16–18). In five cases, inverted papilloma was studied with ISH, and in two cases with a combination of ISH and PCR assay. None of the cases evaluated with ISH were confirmed to be HPV-positive and HPV DNA type 6 was identified by PCR assay in one of the two cases in which it was used (18). The results of the current study are consistent with these ISH results and as a sensitive and wide-spectrum of HPV testing was conducted and found no evidence for the presence of HPV, we conclude that HPV infection is not involved in the occurrence of middle ear squamous papilloma.

There were certain limitations to the examination of HPV in the present study, the most relevant of which was that the sensitivity of ISH appeared to be low compared with that of liquid-phase amplification (10). Thus, if HPV exists in the tissues at low levels, the virus may escape detection by the ISH methods used in the current study. More sensitive methods, including type-specific PCR for HPV, are required in future experiments to determine any role for HPV in the pathogenesis and behavior of middle ear squamous papilloma. Furthermore, although the ISH experiments performed in the present study yielded negative results for HPV infection, there is the possibility of infection with other HPV types. The wide-spectrum probe used detects a number of HPV types simultaneously, including the main types of HPV that have been reported in a variety of head and neck papillomas. However, it does not cover HPV types 2, 3, 10 and 28, which have been shown to be present in other specific cutaneous lesions (19).

EBV is a virus of the herpes family that infects the majority of the human population. This virus infects B cells of the immune system and epithelial cells. There is evidence that infection with EBV is associated with a higher risk of particular forms of cancer and may function as an oncogenic agent (20). Thus, the association of EBV with premalignant changes, including those observed in the four patients of the current study, is worthy of investigation. Furthermore, previous studies have shown that ≤68% of inverted Schneiderian papillomas have tested positive for the presence of EBV (7) and it has been reported that specific cases of non-inverted-type papilloma are also associated with EBV (21). These observations indicate that EBV may be a causative agent of papillomas, but, at present, no results are available with regard to the presence of EBV in middle ear squamous papilloma. In the current study, the squamous papilloma samples obtained from the patients tested negative for EBER despite the notion that EBER ISH is more sensitive than other tests for detecting EBV in tissues (22). Therefore, it is hypothesized that EBV is not associated with the pathologies of this neoplasm.

The symptoms of middle ear squamous papilloma resemble other neoplasms involving the middle ear, including chronic otitis media with cholesteatoma or granulation tissue. Otorrhea and hearing loss are the most common complaints and the majority of cases, including the two previously reported and four present cases, have a clear history of otorrhea with antibiotic treatment that did not resolve symptoms and generally, worsened with time. This is consistent with the assumption that middle ear mucosa metaplasia, due to chronic inflammation, also induces the development of squamous papilloma (2). This tumor may be detected by CT or surgical observations, but an accurate diagnosis must be made pathologically. As squamous papilloma has specific histological features, it must be easily distinguishable from other soft tissue neoplasms in the middle ear, including cholesteatomas and adenomas. Therapy for this tumor is mainly by surgery or biopsy prior to surgery and intraoperative frozen-section examination is currently recommended to exclude malignant disease. Other papillomas in the head and neck areas have a high recurrence rate and the possibility of malignant transformation if not completely excised, therefore, middle ear squamous papilloma has also been predicted to have this trend. Radical tympanomastoidectomy or temporal bone resection must be considered as the initial treatment to guarantee radical resection of the lesion.

Notably, middle ear squamous papilloma in the present cases occurred predominantly in males between the fifth and seventh decades of life, which is similar to the demographic features of head and neck papillary squamous cell carcinoma (23,24). Papillomas associated with premalignant changes were found in two cases of the present study, but definite squamous cell carcinoma was not observed in any of the cases. Therefore, we conclude that this tumor has a tendency for malignant transformation. By contrast, the association of sinonasal inverted papilloma with squamous cell carcinoma is well recognized and varies between 5 and 53%, with an overall average value of 10% (25). It is difficult to obtain definitive information from the literature with regard to the incidence and prognosis of middle ear squamous papilloma associated with carcinoma due to the rarity of this tumor. To the best of our knowledge, the cases presented in the current study are the first reported of this tumor accompanied with premalignant changes. For these patients, radical surgery was sufficient and there was no requirement for postoperative irradiation. In the follow-up periods of >2 years, no recurrence was observed. In spite of this, long-term postoperative follow-up for middle ear squamous papilloma cases is recommended for the purposes of detecting recurrence and monitoring for malignant transformation. As two cases in the present study had a long history of otorrhea, we hypothesize that chronic inflammatory stimulation, not HPV and EBV infection, is a causative agent of premalignant changes. It is entirely possible, however, that infection by other viruses or specific unidentified causes may also be involved in the occurrence of malignant transformation.

In the present study, four cases of middle ear squamous papilloma have been reported. Although this tumor is exceedingly rare, it is possible that its incidence is under recognized. Commonly, these papillomas develop in males of ~60-years of age and otorrhea is the most frequent complaint. Premalignant changes were observed in two of the present cases and ISH of HPV and EBV in all four cases was found to be negative. The present results indicate that chronic inflammatory stimulation, not HPV and EBV infection, is involved in the occurrence of this tumor and its malignant transformation. Radical surgery and long-term postoperative follow-up are recommended due to its malignant and recurrent potential. The current study was limited to a small number of cases and the etiology of this disease remains unclear. Further genetic investigations with newly identified cases are required to clarify the pathogenesis of this disease.

## Figures and Tables

**Table I:** Clinical observations of middle ear squamous papilloma.

Case no. (year)	Gender	Age, years	Presenting symptoms	Localization	Premalignant change	Treatment	Follow-up, months
1 (2009)	M	70	Otorrhea, HL and otalgia	Left middle ear, extending into the inner ear and EAM	+	Subtotal temporal bone resection	NED, 36
2 (2010)	M	50	Otorrhea, otalgia and aural fullness	Left middle ear	+	Partial temporal bone resection	NED, 28
3 (2012)	M	29	Otorrhea, HL and tinnitus	Left middle ear, extending into the EAM	−	Radical tympanomastoidectomy	NED, 12
4 (2012)	M	60	HL, otalgia and tinnitus	Right middle ear, extending into the EAM	−	Radical tympanomastoidectomy	NED, 6

M, male; HL, hearing loss; EAM, external auditory meatus; NED, no evidence of disease.
